# Editorial: Effort-based decision-making and cognitive fatigue

**DOI:** 10.3389/fnins.2023.1230022

**Published:** 2023-06-23

**Authors:** Michel Audiffren, Rémi L. Capa, Nicolas Silvestrini, James Steele, Sabrina Ravel, Benjamin Pageaux

**Affiliations:** ^1^Centre de Recherches sur la Cognition et l'Apprentissage, Université de Poitiers, CNRS, Poitiers, France; ^2^Centre d'études et de recherche en psychopathologie et santé, Institut national universitaire Champollion, Albi, France; ^3^Geneva Motivation Lab, Faculty of Psychology and Educational Sciences, University of Geneva, Geneva, Switzerland; ^4^Faculty of Sport, Health and Social Sciences, Southampton Solent University, Southampton, United Kingdom; ^5^Institut de Neurosciences de la Timone, Aix-Marseille Université, CNRS, Marseille, France; ^6^École de Kinésiologie et des Sciences de l'Activité Physique, Faculté de Médecine, Université de Montréal, Montréal, QC, Canada; ^7^Centre de Recherche de l'Institut Universitaire de Gériatrie de Montréal, Université de Montréal, Montréal, QC, Canada; ^8^Centre Interdisciplinaire de Recherche sur le Cerveau et l'Apprentissage, Montréal, QC, Canada

**Keywords:** effortful control, mental effort, perception of effort, self-control, willpower

## Introduction

During their daily life, humans and animals must frequently make effort-based decisions about choosing among several more or less effortful activities, stopping or maintaining an ongoing effortful activity. In the field of effort-based decision-making, several questions remain to better understand how individuals engage or persevere in cognitive or physical tasks requiring effortful control. This Research Topic aimed to address several questions, such as: How long does it take to recover from cognitive fatigue? How can self-control and boredom interact with exercise behavior? Which brain structures support effortful control? Do perceived exertion scales effectively monitor effort commitment? Is it possible to train willpower?

In this perspective, researchers with interests in psychology, neuroscience and movement science submitted new reflections, data and modeling allowing significant advancements in the understanding of effort-based decision-making and cognitive fatigue in symptomatic and asymptomatic populations. Twenty articles were included in the Research Topic and contributed to answering the abovementioned questions.

The Research Topic is organized into five parts. The first part focuses on the acute effect of cognitive fatigue on motor control and executive control. The second part debates on self-control depletion. The third part is related to effortful control deployment in humans and rats. The fourth part is dedicated to the perception of effort. The fifth part focuses on training programs aiming to improve the capacity to exert effortful control.

## Part 1. Cognitive fatigue

Cognitive fatigue, also known as mental fatigue, is generally observed during or after prolonged engagement in effortful cognitive tasks (Van Cutsem et al., 2017; Brown et al., 2020). Skau et al., the first article of this Research Topic, analyzes different definitions of fatigue and makes a clear distinction between the objective and the subjective manifestations of fatigue. Then, four experimental studies consistently observed a negative impact of cognitive fatigue on performance.

First, Salomone et al. induced cognitive fatigue with a 24-min time load dual back (TLDB) task followed by a 45-min Simon task. Cognitive fatigue was measured through performance indexes as a function of time-on-task during the Simon task. The results showed that time-on-task impaired online control by disrupting the capacity to suppress the incorrect response.

Second, Jacquet et al. demonstrated that cognitive fatigue induced by a 32-min TLDB task negatively impacts the performance in a subsequent arm-pointing task. Concerning the persistence of cognitive fatigue after the TLDB, task performance in the pointing task and theta and alpha power density of brain oscillations recorded during rest periods suggested that participants remained mentally fatigued until 20 min after the end of the cognitive task.

Third, Walker et al. examined the effect of cognitive fatigue in healthy controls and a sample of multiple sclerosis (MS) patients characterized by high fatigability. Cognitive fatigue was induced with different versions of the paced auditory serial addition test (PASAT). Their results showed that the 3-s intertrial interval version of the PASAT is the most effective version to detect impaired performance and cognitive fatigue in MS patients.

Fourth, Wylie et al. used a *n*-back task to induce cognitive fatigue. They measured cognitive fatigue with visual analog scale, indexes from signal detection theory and functional magnetic resonance imaging. They showed that signal detection theory indexes and level of activation in the caudate nucleus significantly correlated with subjective fatigue.

Globally, the four experimental studies of this part showed an impaired performance during or after an effortful task tapping executive functions. Interestingly, two studies (Jacquet et al.; Wylie et al.) support that an increase in activation of the insula and prefrontal theta density could be two biomarkers of cognitive fatigue (Borghini et al., 2014; André et al., 2019).

## Part 2. Self-control failure

**Figure 1:**
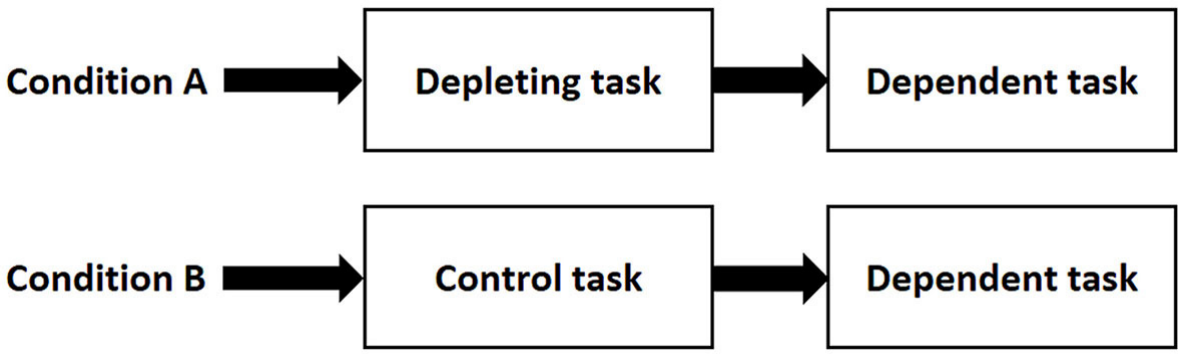
Time course of the sequential task protocol. In condition A, participants perform a depleting task requiring self-control followed by a dependent task also requiring self-control. In condition B, participants perform a control task that does not require self-control followed by the same dependent task than in condition A. The two conditions can be arranged in a within-subjects design (same participants performing two counterbalanced sessions) or in a between-subjects design (one group per condition).

Social psychologists used the sequential task protocol to obtain self-control failure (Figure 1; Lee et al., 2016). This phenomenon, also called ego depletion, is observed after an initial act of self-control and leads to impaired performance in a subsequent self-control task (Dang, 2018).

Over the past decade, the existence of the ego-depletion effect has been challenged by meta-analytic studies (Carter et al., 2015) and faces a replication crisis (Hagger et al., 2016; Vohs et al., 2021). First, Englert and Bertrams present a short history of this replication crisis and criticize the scientific approach used by the replication studies. More importantly, the authors ask for the necessity to clearly operationalize the central constructs of the theoretical model that hypothesize the ego depletion effect to test its validity.

Another problem related to the use of the sequential task protocol, is that the depleting task and the control task can induce boredom and interact with self-control (e.g., Mangin et al., 2021). In this perspective, Wolff et al. suggested that boredom might have contributed to the inconsistencies observed in replication studies by acting as a confound of self-control effects on performance.

As mentioned above, the duration of the depleting task (see Figure 1) seems to be an important parameter in the occurrence of the ego depletion effect. In the third article, Boat et al. manipulated the duration of the depleting task (4, 8, and 16 min). They showed that the performance of the subsequent wall-sit task was more negatively impacted when participants spent longer on the initial self-control task.

In contrast, the fourth and fifth articles (Englert, Dziuba, Giboin et al.; Englert, Dziuba, Schweizer et al.) failed to find an ego depletion effect.

In the sixth article, Alquist et al. presented three experiments using the sequential task protocol and showed that uncertainty introduced in the depleting condition impaired a subsequent task involving executive control. They suggest uncertainty is a cue for conserving effort.

Overall, this section shows that there are still strong debates about the conditions of occurrence of the ego depletion effect.

## Part 3. Effort-based decision making

The third part is dedicated to the decision-making process allowing the deployment of effortful control according to the costs and benefits associated with the achievement of the goal of the task. Cost-benefit models (e.g., Shenhav et al., 2017) are presently the most popular models of effort-based decision-making in economics, neuroscience and psychology. These models consider that an individual consent to deploy effort when benefits outweigh costs. Numerous neurophysiological studies suggest that the anterior cingulate cortex (ACC) plays a crucial role in this decision-making process (André et al., 2019; Müller and Apps, 2019). This part includes five articles that contribute to a better understanding of this process.

The first study conducted by Jiang et al. aimed to examine the deployment of effortful control with event-related potential (ERP) in schizophrenia, a mental disorder often associated with deficits of effort mobilization (McCarthy et al., 2016). Their results suggest that schizophrenia patients experienced an increased mental workload and slowed processing speed due to effortful information processing deficits.

In the second article, Lacroix et al. introduced a neuropsychological model based on Kahneman's capacity model of attention (Egeth and Kahneman, 1975). Their model partially explains the variability of results observed in vestibular-damaged patients and contributes to the understanding of the vestibular compensation process.

In the third article, Silva et al. investigated whether electrical stimulations of the ACC or anterior insula, change the rat's persistence in an effortful weightlifting task. Their results confirm the crucial role of the ACC in the deployment of effortful control during a cognitive or physical task.

In the fourth study, van As et al. examined whether individuals weigh physical effort-costs more strongly when they are cognitively or physically fatigued. For that purpose, they induced cognitive fatigue with a 45-min 2-back task or physical fatigue with a 45-min intermittent submaximal handgrip task. In the subsequent effort-based decision-making task, participants had repeatedly to accept or reject offers with varying levels of rewards and physical effort. The results of this study suggest that individuals ascribe more weight to physical effort-costs than cognitive effort-costs.

In the last study of this part, Feng et al. aimed to validate a model of procrastination with mathematical simulations. Their model predicts that procrastination can be mitigated by explicitly informing an individual about the remaining future cost associated with procrastination and the possible decrement of this cost if the individual chooses to perform the procrastination-related behavior.

## Part 4. Perception of effort

Previous research in sports sciences showed that the perception of effort in a physical task, also called perceived exertion, is increased when it is preceded by a long cognitive effortful task (Marcora et al., 2009; Pageaux and Lepers, 2016). The two experimental studies included in this part aimed to validate the use of the perception of effort to prescribe and/or monitor exercise in healthy young adults.

In the first article, Payen de la Garanderie et al. conducted two experiments to manipulate the physical demand and alter the difficulty of the task. They monitored the perception of effort with the Borg's CR100 scale while controlling for performance in two upper limb motor tasks: the box and block test and a pointing task. The authors showed that perception of effort is a valid tool to prescribe and monitor exercise during upper-limb motor tasks.

In the second article, Armes et al. examined the validity of repetitions in reserve (RIR) scales that are used to assess and/or control effort by participants estimating how many repetitions they can perform before reaching momentary task failure during resistance exercises. They conducted two experiments to test the validity of the RIR scales in resistance exercises with submaximal intensities. They showed that participants with at least 1 year of resistance training experience are likely not adequately accurate at gauging effort in submaximal conditions. These results suggest that the RIR scale during resistance training exercise may not be as accurate as needed to estimate accurately the actual effort.

These two studies emphasize the importance for researchers to carefully check in the literature the validity of the psychophysical scales they plan to use for assessing effort allocation, as not all scales seem adequate. Indeed, while one was able to monitor quite accurately the effort deployed in fine motor tasks, the second one was inadequate to predict task failure in submaximal resistance exercises.

## Part 5. Training the capacity to deploy effortful control

This part includes two articles dedicated to the hypothesis of improving the capacity to exert effortful control in difficult conditions through training programs. In this perspective, two recent studies tested the efficacy of brain endurance training programs and received relative success (Dallaway et al., 2021, 2023).

First, Audiffren et al. report an umbrella review that examined the efficacy of different training programs in improving executive functions and self-control. The results of 63 meta-analyses on this topic were analyzed. More than 79% of these reviews showed that training programs are effective in improving performance in tasks tapping executive functions and/or self-control with a small to large effect size. Training programs including physical exercises or mindfulness exercises seem to be the most promising in terms of far-transfer effects. In the second part of the article, the authors propose a theoretical neuroscience framework explaining these gains in willpower.

In the second article, Holmqvist et al. showed that patients with mild to moderate stroke or traumatic brain injury can benefit from a 6-week intensive cognitive training. The training program targeted five attention modalities: focused, sustained, selective, divided, and flexible attention. Their results suggest that patients with high levels of cognitive fatigability benefit most from attention training.

The two articles of this part suggest that training the capacity to maintain effortful control despite internal or external constraints is a promising way to increase the resistance to cognitive fatigue. The development of effective training programs applied to specific domains (e.g., sport, education, labor) is a very exciting perspective.

## Conclusion and perspectives

The investigation of effort-based decision-making and cognitive fatigue embraces several scientific disciplines, such as economics, psychology, neuroscience, and exercise physiology. A better understanding of these two scientific objects requires at least a multidisciplinary, and more optimally an interdisciplinary approach. This Research Topic constitutes an additional step in this direction, and we hope that the reading of this selection of articles has significantly contributed to the advancement of this scientific field.

## Author contributions

MA wrote the manuscript. RC, JS, NS, SR, and BP revised and edited the manuscript. All authors contributed to the article and approved the submitted version.
